# Heterogeneity of ILC2s in the Lungs

**DOI:** 10.3389/fimmu.2022.918458

**Published:** 2022-06-09

**Authors:** Masato Asaoka, Hiroki Kabata, Koichi Fukunaga

**Affiliations:** Division of Pulmonary Medicine, Department of Medicine, Keio University School of Medicine, Tokyo, Japan

**Keywords:** group 2 innate lymphoid cells, heterogeneity, development, plasticity, memory, aging

## Abstract

Group 2 innate lymphoid cells (ILC2s) are GATA3-expressing type 2 cytokine-producing innate lymphocytes that are present in various organs throughout the body. Basically, ILC2s are tissue-resident cells associated with a variety of pathological conditions in each tissue. Differences in the tissue-specific properties of ILC2s are formed by the post-natal tissue environment; however, diversity exists among ILC2s within each localized tissue due to developmental timing and activation. Diversity between steady-state and activated ILC2s in mice and humans has been gradually clarified with the advancement of single-cell RNA-seq technology. Another layer of complexity is that ILC2s can acquire other ILC-like functions, depending on their tissue environment. Further, ILC2s with immunological memory and exhausted ILC2s are both present in tissues, and the nature of ILC2s varies with senescence. To clarify how ILC2s affect human diseases, research should be conducted with a comprehensive understanding of ILC2s, taking into consideration the diversity of ILC2s rather than a snapshot of a single section. In this review, we summarize the current understanding of the heterogeneity of ILC2s in the lungs and highlight a novel field of immunology.

## Introduction

Group 2 innate lymphoid cells (ILC2s) express transcription factor GATA3 and produce type 2 cytokines upon stimulation with epithelial cell-derived cytokines, including IL-33 and IL-25. ILC2s, which were first characterized by three different research groups in 2010 (1–3), are distributed throughout the body, such as in the lungs, skin, intestine, liver, brain, bone marrow, and peripheral blood in mice and humans (1–13). In particular, ILC2s play a critical role in innate immunity-mediated type 2 airway inflammation (1–8, 10–12) and contribute to the repair of airway damage *via* amphiregulin production after influenza virus infection (14). Recent studies have shown that ILC2s are associated with lung fibrosis, chronic lung obstructive disease exacerbation, and lung cancer (15–23). Thus, ILC2s have various functions and are involved in the pathogenesis of several lung diseases.

ILC2s are primarily tissue-resident cells with different characteristics depending on the tissues in which they exist (24–27). In mice, ILC2s express higher levels of *Il1rl1*, which encodes the IL-33 receptor subunit ST2, in the lungs than in other tissues (27, 28). Conversely, the expression levels of *Il17rb*, which encodes an IL-25 receptor subunit, and *Il18r1*, which encodes an IL-18 receptor subunit, are lower in ILC2s in the lungs than in the small intestine and skin, respectively (27). Indeed, intranasal administration of IL-33 potently activates ILC2s in the lungs compared with IL-25 or IL-18 (27, 29, 30). Interestingly, when IL-25 is administered intraperitoneally (but not intranasally), an intestine-derived unique ILC2 subset (called inflammatory ILC2s [iILC2s]), which highly expresses KLRG1 and IL-17RB, migrates to the lungs (31). Moreover, recent single-cell RNA-seq analyses in mice and humans have revealed the existence of diverse cell populations among lung ILC2s (27, 32–38). Although ILC2s were previously considered to be a single-cell population, these results confirm the presence of different subsets within ILC2s. Therefore, it is necessary to dissect individual cell populations to evaluate their molecular mechanisms and relationships with diseases.

In this review, we provide a current overview of the heterogeneity of ILC2s in the lungs, and summarize our understanding of the diversity of ILC2s and their contribution to immunity in the lungs.

## Differences in ILC2s Across Tissues and Heterogeneity of Lung ILC2s at Steady State

**Figure 1:**
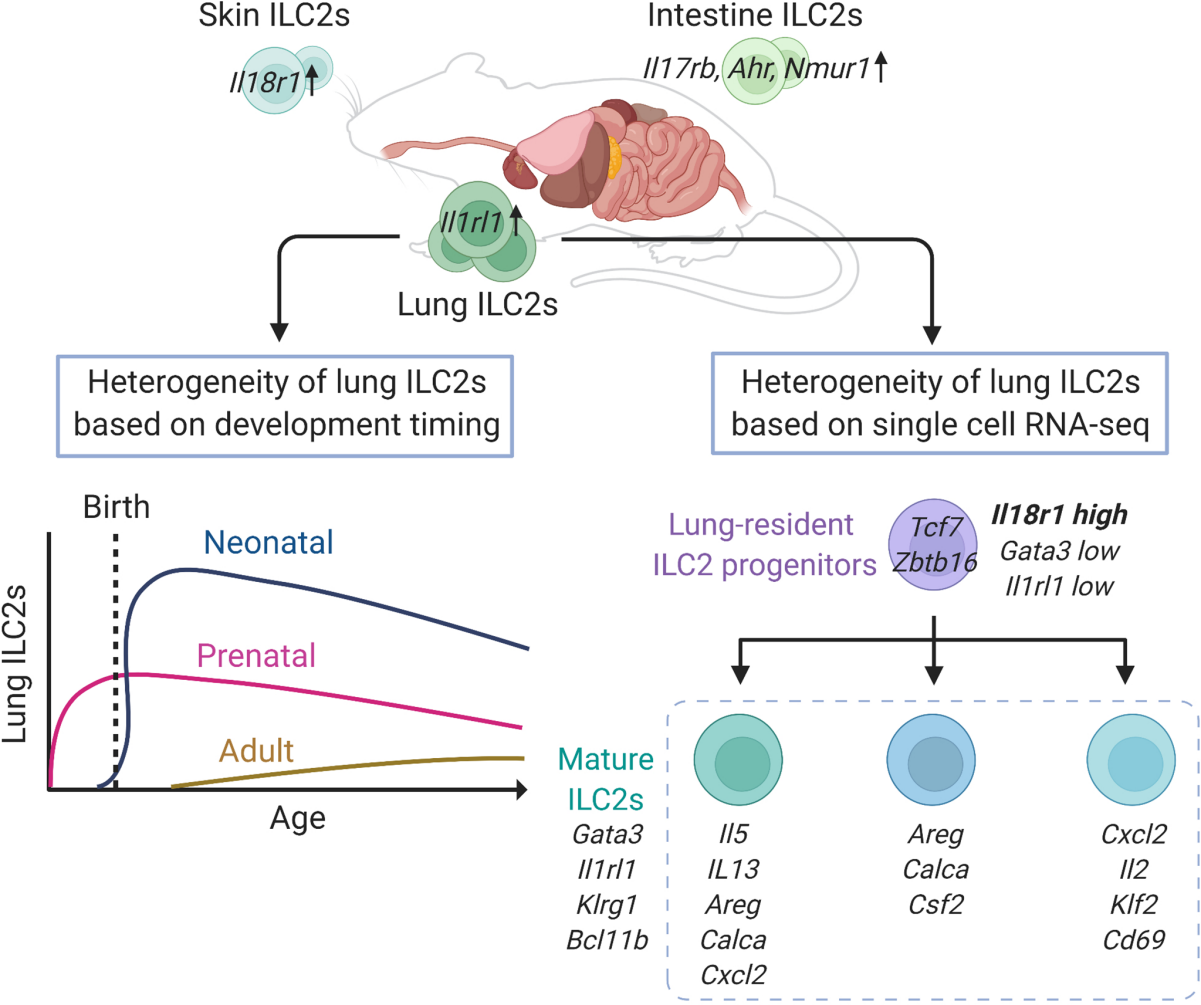
Heterogeneity of lung group 2 innate lymphoid cells (ILC2s) in mice at steady state. Lung ILC2s have increased expression of *Il1rl1*, while intestinal ILC2s highly express *Il17rb*, *Ahr* and *Nmur1*, and *Il18r1* is enriched in skin ILC2s, respectively. Adult ILC2s in the lung have three developmental origins: prenatal, neonatal, and adult origin. The lung resident-ILC2 progenitor cells differentiate into at least three subtypes of mature ILC2s. This figure is created based on ref (27, 32, 41).

ILC2s are distributed in various organs of the body at a steady state. A recent study comparing the expression of various genes in murine ILC2s in various tissues, such as lung, skin, adipose, skin, intestine, and bone marrow, showed that the expression levels of *Gata3* and *Il7r* do not differ among ILC2s in each tissue; however, hundreds of genes are differentially expressed by ILC2s depending on the localized tissue (27). In particular, lung ILC2s display increased expression of *Il1rl1*, while intestinal ILC2s highly express *Il17rb* as well as *Ahr* and *Nmur1*, which encode aryl hydrocarbon receptor (AHR) and neuromedin U receptor 1 (NMUR1), respectively (27, 38, 39). Indeed, intranasal administration of IL-33 potently activates lung ILC2s compared with IL-25, while genetic ablation of *Ahr* or *Nmur1* modifies anti-helminth immunity *via* intestinal ILC2s (38, 39). In addition, most skin ILC2s that express lower levels of *Il1rl1* and *Il17rb* also show increased expression of *Il18r1*, which encodes an IL-18 receptor subunit (27). Interestingly, the tissue-specific features of ILC2s are independent of either the microbiome or epithelial cell-derived cytokines, including IL-25, IL-33, and thymic stromal lymphopoietin (TSLP). However, neuropilin-1 (*Nrp1*) was recently identified as a candidate gene that determines the tissue specificity of lung ILC2s and enhances the expression of IL-33 receptor (40). NRP1 can be induced postnatally and its expression is maintained in the lung environment by TGF-β signaling, resulting in the establishment of tissue specificity by lung ILC2s (40). These findings suggest that the diversity of ILC2s depends on the tissue in which they reside, and that acquisition of tissue specificity may begin very early in development (**Figure 1**).

During the development of ILC2s in the lungs, ILC progenitors migrate to the lung before birth and differentiate into ILC2s depending on IL-7 signaling (41). These prenatal ILC2s persist after birth, and are detectable in adult mice, however, they account for a small proportion and decrease further with age. After birth, acute expansion and activation of ILC2s occur in the lungs between 2 and 3 weeks of age, and these neonatal ILC2s have high expression of genes related to cytokine production, immunoregulation, and proliferation, such as *Il5*, *Il13*, *Nr4a1*, and *Mki67*, as well as *Cxcl2*, a neutrophil recruitment factor, which induces other immune cells into the lung and contributes to the development of the lung environment (41, 42). According to the fate-mapping approach, neonatal ILC2s are long-lived and tissue-resident and constitute the majority of lung ILC2s, even in adults (41). In adult mice, the proportion of neonatal ILC2s is gradually diluted by newly generated ILC2s, termed adult-derived ILC2s, and the adult lungs contain a diverse mixture of prenatal, neonatal, and adult-derived ILC2s. However, the turnover speed of ILC2s varies depending on the tissue and is slower in the lungs than in the skin, small intestine, and bone marrow. Therefore, the proportion of neonatal ILC2s is high in the lungs, and neonatal ILC2s play a major role in enhancing type 2 inflammation through local expansion in the lungs (41).

To assess the diversity of lung ILC2s at a steady state, a study performed single-cell RNA-seq analysis of lung ILC2s from adult mice, which revealed that lung ILC2s can be divided into several subgroups, including a group that highly expresses *Cxcl2*, *Il2*, *Klf2*, and *Cd69*; a group that expresses *Areg*, *Calca*, and *Csf2*; and a group that expresses *Il5*, *Il13*, *Cacla*, *Areg*, and *Cxcl2* (32). However, it is not clear whether these are groups of cells at different developmental stages or activation states. This study also identified a small number of ILC progenitors with low expression of *Gata3* and *Il1rl1*, and high expression of *Il18r1* in the lungs (32). These cells express ILC progenitor marker genes, such as *Tcf7* and *Zbtb16*, as well as genes that suggest a mixed lineage potential, such as *Rorc* and *Tbx21*. Although these cells account for less than 2% of ILCs (Lin-IL7R+ cells), trajectory analysis suggests that these cells lose progenitor markers, such as *Cd7*, *Il18r1*, *Tcf7*, and *Zbtb16*, gradually increase the expression of *Gata3*, *Il1rl1*, *Klrg1*, and *Bcl11b*, and differentiate into mature ILC2s (**Figure 1**). Therefore, these data suggested that local ILC progenitors differentiated and matured towards ILC2s within the lung tissue (32).

While ILC2s are the dominant population of ILCs in murine lungs, percentage of ILC2s among lung-resident innate lymphocytes in human is around 30% (13, 35, 36, 43). Human ILC2s express *IL1RL1*, *IL17RB*, *KLRG1*, *GATA3*, and *PTGDR2*, which encode CRTH2, however, there is diversity in the expression of various genes depending on the localized tissues (33–36, 44). Specifically, human lung ILC2s show higher expression levels of *IL1RL1*, *IL17RB*, and *IL13* than tonsil and blood ILC2s (34–36). In addition, *SLAMF1*, *TNFRSF9*, *FFAR3*, and *PPARG* expression are upregulated in human lung ILC2s. SLAMF1 (CD150), encoded by *SLAMF1*, belongs to the signaling lymphocytic activation molecule (SLAM) family that modulates the activation of immune cells, including T cells (34, 45). *TNFRSF9* encodes the activation-induced surface receptor TNFRSF9, which was originally found in activated T cells (34, 46). *FFAR3* and *PPARG* are lipid metabolism-related genes that regulate the immune cells (47–49). Therefore, the upregulation of these four genes implies a tendency for cell activation. In contrast, human blood ILC2s show high expression levels of *PTGDR2*, *S1PR2*, and *CCR2*, which are migration markers (33, 34). While CRTH2 is one of the representative surface markers of human ILC2s, the expression of CRTH2 in blood ILC2s is downregulated after stimulation with a combination of IL-2, IL-25, IL-33, and TSLP, suggesting that CRTH2 expression is negatively correlated with ILC2 activation (34). Among lung ILC2s, approximately 35% of ILC2s lack CRTH2 expression (34). Based on these findings, human lung ILC2s may be relatively activated even in the steady state.

Although the diversity within human lung ILC2s has not been well studied due to their small number, a human study of single-cell RNA-sequence analysis using fetal samples following elective medical termination of pregnancy has been recently reported (33). In this study, fetal lung ILC2s were divided into five subgroups: Pre-ILC2s, CRTH2_ILC2s, PTGS2_ILC2s, CCR9_ILC2s, and KIT_ILC2s. Pre-ILC2s highly express *PRSS57* and *SPINK2*, which are associated with stem cell signaling and are suggested to be ILC2 progenitors. Pre_ILC2s were further divided into two subgroups according to the expression levels of *MKI67, PTGDR2*, and *BCL11B*, indicating that immature Pre_ILC2s differentiate into restricted ILC2 progenitors, thereby upregulating the expression of these three genes. CRTH2_ILC2s and PTGS2_ILC2s had high expression levels of *PTGDR2*, and can be considered as conventional ILC2s. In particular, CRTH2_ILC2s exhibited the highest expression levels of *IL1RL1* and *IL13*, and PTGS2_ILC2s expressed high levels of *PTGS2*, which encodes COX2. CCR9_ILC2s and KIT_ILC2s are unconventional ILC2s because of low expression levels of *PTDGR2*. CCR9_ILC2s are a rare population of lung ILC2s that express T cell marker genes, including *CD1E*, *CD2*, *CD3G*, *CD4*, and *CD8A*; however, their biological roles are unknown. KIT_ILC2s express *CCR6* and *LTB* and show plasticity, converting into IL-17^+^ILC3-like cells, suggesting that KIT-ILC2s may be involved in ILC2 plasticity. Thus, it has been reported that there is diversity in the lung ILC2s of human fetuses; however, the relationship to disease and diversity in adults has not yet been determined.

## Heterogeneity of Activated ILC2s in the Lungs

Various stimuli, such as allergens and viruses, induce the release of epithelial cell-derived cytokines, including IL-25 and IL-33. These cytokines enhance the phosphorylation of GATA3 *via* the NF-κB and MAPK signaling pathways in ILC2s (50), which induces cell proliferation and production of type 2 cytokines, such as IL-5 and IL-13. Although murine lung ILC2s express CD25, CD90.2, CD127, KLRG1, Sca-1, and ST2, the expression levels of these surface markers vary depending on the mouse strain and sex. Furthermore, they vary largely with the type of stimulation; intranasal administration of IL-33, house dust mite, or *Alternaria alternata* extract induced the different expression levels of surface markers, including CD25 and KLRG1, depending on the stimuli (51). In addition, a recent study evaluated the diversity of activated lung ILC2s in mice treated with IL-33 or IL-25 using single-cell RNA-seq analysis and flow cytometry (38). Intranasal administration of IL-25 or IL-33 increased the expression of *Il5*, *Il13*, *Klrg1*, *Arg1*, and *Areg* genes, as well as *Gp49* and *Batf*, and some subsets of ILC2s also increased MHC class 2 and CTLA4. Interestingly, *Nmur1* was highly expressed in lung ILC2s at the steady state and after IL-25 stimulation; however, it was downregulated by IL-33. Furthermore, the expression of semaphorin 4a was reduced by IL-33 stimulation. These results suggest that activated ILC2s can alter the expression of various genes and surface markers, depending on the stimulus.

*Nippostrongylus brasiliensis* (*N. brasiliensis*) causes migratory helminth infection in mice. Within hours of entry through the skin, *N. brasiliensis* stage 3 larvae migrate *via* the blood stream to the lung and alveolar space, causing local tissue damage and hemorrhage on days 1–2 post infection. Maturing larvae are then transported up the airways and swallowed to take up residence in the small intestine on days 4–7 post infection to allow adult reproduction before eventual expulsion. It has been reported that *N. brasiliensis* infection or an intraperitoneal injection of IL-25 induces a different subtype of ILC2s in the lungs and mesenteric lymph nodes, termed inflammatory ILC2s (iILC2s) (31). iILC2s are undetectable in the lungs at the steady state and have high expression of KLRG1 and IL-17RB; however, they have low expression of ST2 and Sca-1. Notably, iILC2s are circulating cells that arise from gut ILC2s residing in the intestinal lamina propria, and migrate to diverse tissues based on sphingosine 1-phosphate (S1P)-mediated chemotaxis (52). iILC2s contribute to the regulation of anti-helminth immunity by producing type-2 cytokines similar to lung ILC2s, that are called natural ILC2s (nILC2s), to distinguish them from iILC2s. Furthermore, iILC2s express not only GATA3, but also intermediate levels of RORγt, suggesting that iILC2s carry out both nILC2-like and ILC3-like functions. Indeed, iILC2s produce IL-17 after an oral *Candida albicans* infection and contribute to antifungal immunity (31). Recently, IL-33 was reported to promote the generation of iILC2s *via* induction of tryptophan hydroxylase 1 (Tph1). Ablation of *Tph1* resulted in the impairment of iILC2 responses *via* modified expression of ICOS and increased susceptibility to *N. brasiliensis* infection (53).

**Figure 2:**
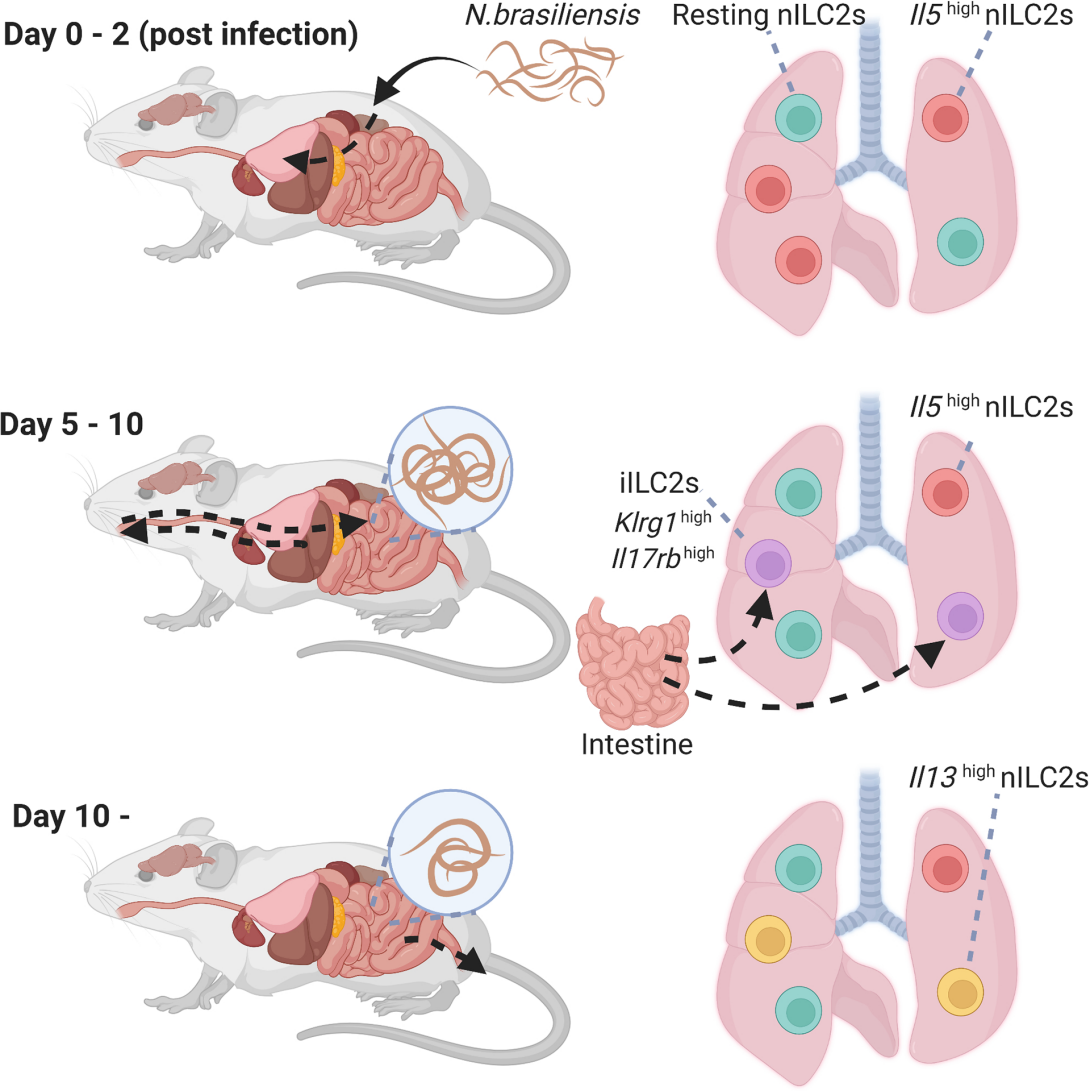
Heterogeneity of lung group 2 innate lymphoid cells (ILC2s) during *N.brasiliensis* infection. Time course of activation of lung ILC2s in the lungs following a helminth infection. High levels of *Il5* ^high^ natural ILC2s that appear shortly after the infection. Then, iILC2 populations transiently increased, and *Il13* ^high^ natural ILC2 populations increased thereafter. iILC2, inflammatory type 2 innate lymphoid cell; nILC2, natural type 2 innate lymphoid cell; *N. brasiliensis*, *Nippostrongylus brasiliensis*.

Two studies investigated the heterogeneity of lung ILC2s during an *N. brasiliensis* infection using single-cell RNA-seq analysis (32, 54). In the first study, lung ILC2s were divided into four groups based on their gene clusters: resting nILC2s, *Il5* ^high^nILC2s, *Il13* ^high^nILC2s, and iILC2s (54). *Il5* ^high^nILC2 populations increased early after the *N. brasiliensis* infection (day 2), however, this proportion gradually decreased. On day 5, iILC2 populations transiently increased and became the major component of lung ILC2s. iILC2s, however, were undetectable on day 9, and *Il13* ^high^nILC2 populations increased thereafter (days 9 and 14) (**Figure 2**). Similarly, another study showed that the populations of ILC2s expressing *Calca*, *Csf2*, and *Cxcl2* increased on day 4 of the *N. brasiliensis* infection, whereas the populations of blood-derived circulating iILC2s expressing *Klrg1* increased on days 7 and 10 (32). On day 15, various subtypes of ILC2s were induced. Interestingly, fate mapping revealed that ILC2s on days 4 and 15 were differentiated from ILC progenitors in the lung rather than being replenished from the bone marrow. Thus, the diversity of ILC2s changes dynamically over time during *N. brasiliensis* infections.

Although there are no studies on the diversity of activated lung ILC2s in humans, a recent study reported that most resting ILC2s in humans express CD45RA; however, there is a large population of ILC2s expressing CD45RO in inflammatory mucosal tissues, including nasal polyps (55). The transcriptomic features of these cells were similar to those of mouse iILC2s, and peripheral blood-derived resting ILC2s expressed CD45RO in response to the stimulation. Thus, CD45RO may be a useful activation marker for human ILC2s.

As described previously, ILC2s produce type 2 cytokines in a GATA3-dependent manner upon stimulation with IL-33 or IL-25; however, their properties are known to be greatly altered by the surrounding cytokine environment. ILCs are divided into three groups according to their transcription factors and functions: ILC1s, ILC2s, and ILC3s; however, ILCs have plasticity, and can acquire the properties of other ILCs.

First, upon exposure to viruses, bacteria, or noxious agents, such as cigarette smoke, murine lung ILC2s convert to an ILC1-like phenotype with decreased expression of GATA3 and IL-33 receptor ST2, increased expression of T-bet, and the ability to produce IFN-γ (18). This conversion to an ILC1-like phenotype is mediated by stimulation with IL-1β, IL-12, or IL-18 (18, 56). Human ILC2s also acquire an ILC1-like phenotype *via* IL-1β, IL-12, and IL-18 (57, 58). Indeed, ILC1 populations are reported to increase in patients with chronic obstructive pulmonary disease (COPD), suggesting that ILC2s may be converted to ILC1s owing to the influence of cigarette smoke (18).

Second, a combination of IL-33 with leukotriene C4 or D4 can induce IL-17-producing ILC2s termed ILC2_17_s in mice. Unlike Th17 cells or ILC3s, ILC2_17_s are independent of RORγt expression (59). ILC2_17_s produce IL-17, IL-5, and IL-13 by an intranasal administration of IL-33 or papain; however, Ahr-deficient ILC2s produce limited amounts of IL-17 since Ahr is essential for the induction of ILC2_17_s. In humans, IL-2, IL-1β, IL-23, and TGF-β stimulation induces peripheral blood-derived ILC2s to convert to C-Kit^+^ NKp44^-^ILC3-like cells that have the ability to produce IL-17 (60).

Recently, IL-10-producing ILC2s termed ILC2_10_s were found in the lungs of mice after an intranasal administration of IL-33 or papain (61). In IL-33-treated mice, ILC2_10_s represent a major proportion of IL-10-producing cells, and ILC2_10_s can suppress activated ILC2s directly *via* IL-10. However, ILC2_10_s also have the ability to produce type 2 cytokines upon stimulation by IL-33 and TSLP. Mechanistically, retinoic acid (RA) and IL-2 induce ILC2_10_s, whereas TGF-β inhibits IL-10 production (62). RA also induces IL-10-producing ILC2s in human ILC2s, suggesting that RA plays an important role in ILC2_10_s generation (61).

Together, ILC2s change their phenotype dramatically depending on the surrounding environment. These phenotypical changes may constitute a part of heterogeneity of ILC2s both in the steady state and activated state. However, while effector function of each phenotypical plasticity *in vitro* was reported, distinct role *in vivo* remained unclear. Therefore, further survey is awaited.

## Heterogeneity of Lung ILC2s Post Inflammation

ILC2s are diverse even after they are activated once. Recent studies have shown that lung ILC2s can acquire immunological memory. In mice, some activated lung ILC2s mediated by an intranasal administration of papain or IL-33 have the ability to induce strong ILC2-mediated inflammation against secondary challenge. These cells exhibit immunological memory properties and are termed “memory-like ILC2s” (63). Memory-like ILC2s do not exhibit antigen specificity and respond strongly to a second stimulus regardless of the allergen type.

Memory-like ILC2s have some genetical similarities with activated ILC2s but they are resting cells unlike activated ILC2s. Memory-like ILC2s have higher expression levels of *Il1r2, Il5, Tnfsf18, Bcl2a1b, Bcl2a1d, Ler3*, *Syne1*, and *Il17rb* compared to those of naïve ILC2s, suggesting that memory-like ILC2s are activated. Since the expression of *Il17rb* is enhanced in memory-like ILC2s, they produce type 2 cytokines upon IL-25 stimulation, whereas naïve ILC2s do not respond to IL-25 stimulation alone (63).

Although *S1pr1, Il6st, Cd2, Cd7*, and *Sell* expression are lower in both activated ILC2s and memory-like ILC2s than naïve ILC2s, the expression of cell cycle-related genes including *Mki67* and *Ccnb2*, and the chemokine genes including *Ccl17, Ccl24, Cxcl3*, and *Ccl6* are lower in memory-like ILC2s compared to that in activated ILC2s, suggesting that memory-like ILC2s do not proliferate or produce chemokines as much as activated ILC2.

On the contrary, repetitive stimulation induces hyporesponsive phenotypes of ILC2s, which are termed “exhausted-like ILC2s” (64). This subset of ILC2s expresses high levels of *Il10* and *Tigit*, which are considered exhaustion markers. In addition, they express higher levels of PD-1, GITR, and KLRG1 than those in naïve ILC2s. In contrast to ILC2_10_s, exhausted-like ILC2s are incapable of producing type 2 cytokines. A previous report showed that exhausted-like ILC2s could be collected from mice intranasally instilled with 100 μg papain every three days (64). On day 7 after the administration of the three papain doses, exhausted-like ILC2s emerged in bronchoalveolar lavage (BAL) fluid only. In addition, papain administration every three days for a month induced the generation of exhausted-like ILC2s both in the BAL fluid and lung. Thus, the intensity or duration of stimulation changes the fate of lung ILC2s from the acquisition of immunological memory to the loss of functional exhaustion.

## Heterogeneity of Lung ILC2s in Aging

Senescence is characterized by the progressive loss of physiological function in individuals with age (65, 66). DNA damage throughout life induces “cellular senescence”, resulting in a poor proliferative capacity and irreversible cell cycle arrest, and results in a proinflammatory senescence-associated secretory phenotype (SASP) that leads individuals to low-grade, chronic inflammatory state termed as “inflamm-aging (67–71)”. In mice, there is a marked increase in the expression of ILC2 progenitors in the bone marrow of aged (19–24 months old) mice compared with that in young (2–3 months old) mice; however, this increase was not observed in their progenitor cells, common helper-innate lymphoid progenitors (CHILPs) (72). Notch signaling could be involved in this increase in ILC2 progenitor expression with age; this pathway is specific to aged mice and is not involved in ILC2 progenitor differentiation in young mice. While the expression levels of ST2 are lower in aged ILC2 progenitors, young and aged ILC2 progenitors have the same expression level of Ki67, indicating that ILC2 progenitors of aged mice preserve the reproductive activity (72). In accordance with the increase in ILC2 progenitor expression in the bone marrow, ILC2 progenitor populations in the peripheral blood and ILC2 populations in the peripheral blood and small intestine also increase with aging. However, since bone marrow ILC2 progenitors are rarely transferred to the lung and lung ILC2s are highly dependent on local expansion of lung-resident ILC2 progenitors, lung ILC2 populations are reduced with aging.

Functionally, aged lung ILC2s show decreased expression of *Gata3*, *Il5*, and *Areg*, and reduced proliferative capacity and cellular function. Indeed, aging mice are susceptible to viral infections; however, transplantation of young mouse-derived ILC2s promotes recovery from viral infections (72). Furthermore, the expression of *Ehhadh* and *Cyp2e1*, which are involved in peroxisome proliferator-activated receptors (PPAR) pathway and cytochrome P450 (CYP) activity, respectively, is downregulated in aged ILC2s, and these genes enhance IL-5 production and Areg expression independent of GATA3 involvement (72). In addition, the levels of IL-12 and IL-18, that are able to suppress Cyp2e1 expression, were increased in the lungs of aged mice, suggesting that both intrinsic and extrinsic mechanisms cause cellular senescence in lung ILC2s (72).

The effects of aging on ILC2s in humans are poorly understood. In contrast to mice, peripheral blood ILC2 populations decrease with age in humans (73). Lung ILC2 populations in humans may also be negatively correlated with aging, similar to murine lung ILC2s (35). However, studies on human lung ILC2s are not sufficiently large to allow the estimation of this trend.

## Discussion

Since the discovery of ILC2s about a decade ago, a large number of studies on ILC2s have been carried out, and these studies have greatly contributed to our understanding of the immunology and unraveling of the underlying pathophysiology of various human diseases. Various molecules and regulators of ILC2s have been reported, and the general features of ILC2s are widely understood. However, ILC2s have received increasing attention in the last few years because they comprise a heterogeneous cell population rather than homogeneous.

Basically, ILC2s are tissue-resident cells, and the expression levels of transcription factors and receptors of ILC2s vary among tissues. Murine lungs consist of ILC2 progenitor cells and mature ILC2s at different developmental time points in the steady state, and tissue specificity of ILC2s develops postnatal due to the lung tissue environment. Lung mature ILC2s are not only activated by IL-33 due to the high expression of IL-33 receptors, but also by other stimuli, including IL-25 and allergens. However, besides “activation,” changes in the expression of surface antigens and transcription factors vary depending on the stimulus that “activate” ILC2s, and in particular, migratory helminth infection or an intraperitoneal administration of IL-25 transiently induces intestinal-derived iILC2s in the lungs.

**Figure 3:**
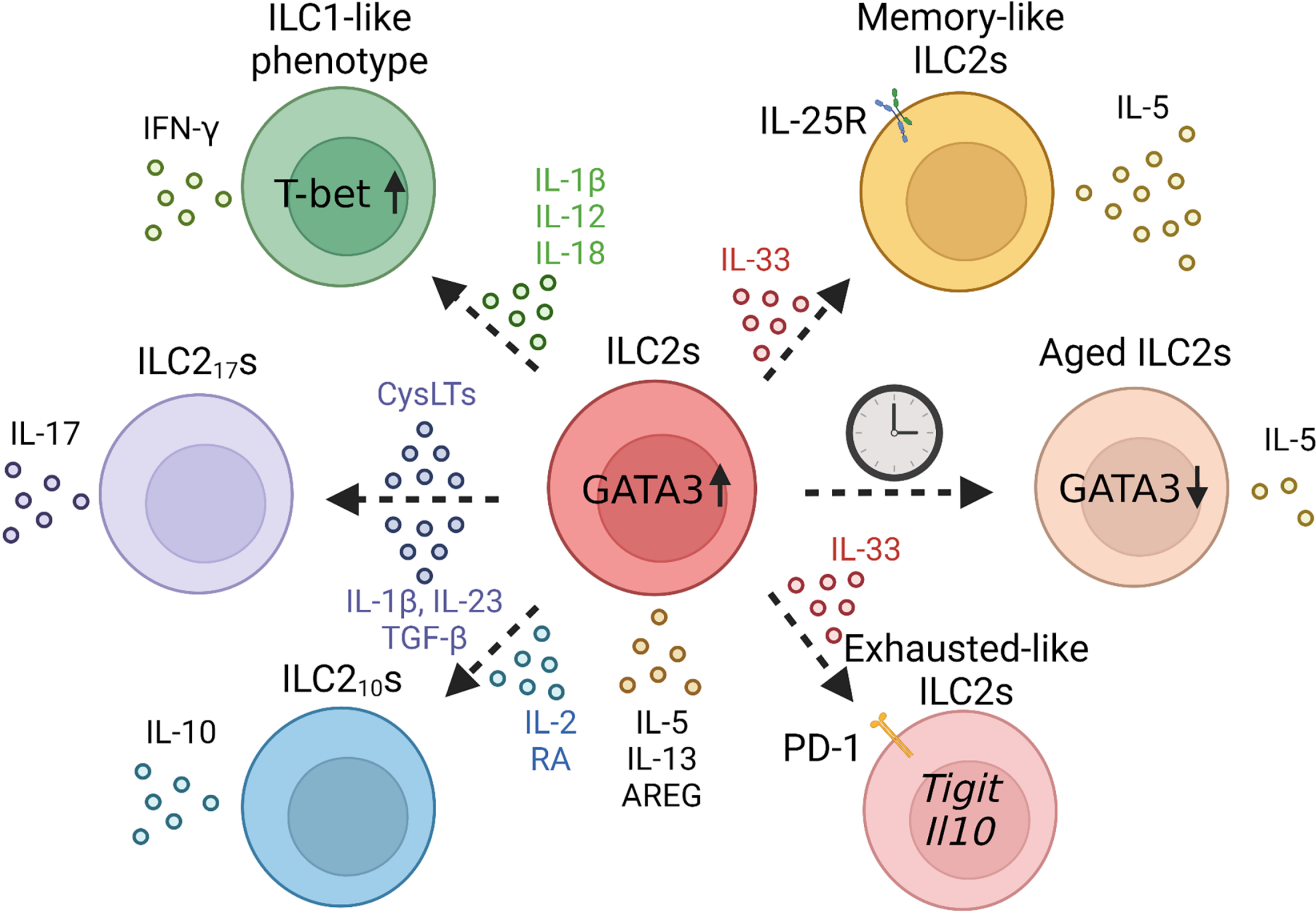
Overview of plasticity and trained lung group 2 innate lymphoid cells (ILC2) in the lung.

Recently, it has been reported that ILC2s have plasticity, and their properties are variable, such as the production of IFN-γ, IL- 10, and IL-17, depending on the surrounding environment, which also contributes to the diversity of ILC2s. While the plasticity is important and a well-known concept of ILC2s, its immunological role is unclarified. Moreover, boundaries between the concept of plasticity and heterogeneity of ILCs are unclear. Furthermore, after activation, ILC2s develop training immunity; however, they sometimes cause cell exhaustion. These diversities change with the aging time scale, and cellular senescence is induced by intracellular and extracellular factors in the lungs (**Figure 3**).

Despite the efforts of many studies to elucidate the diversity of lung ILC2s, several questions remain unclear: Does the diversity of lung ILC2s play a distinct physiological role *in vivo*? If so, what would happen if lung ILC2s are homogenous and lack plasticity? Does the transcriptional heterogeneity of ILC2s, especially at steady state have a unique biological role or does it merely reflect differences in mRNA expression? Additionally, does each ILC2 subtype affect lung immunity and have clinical significance in humans?

Although human studies are also insufficient, the studies summarized in this review contribute to our understanding of disease conditions in humans. The acquisition of immunological memory may contribute to allergen-specific asthma exacerbation and non-specific allergic inflammation. Exhaustion and aging of ILC2s may limit the type 2 inflammation response, especially in geriatric patients, because these changes attenuate the effector function of ILC2s. Therefore, a greater understanding of ILC2 subsets may provide new insights into the pathophysiology of lung diseases, including asthma. In addition, targeting specific ILC2 subsets may provide a new therapeutic strategy for lung diseases.

To date, most studies have only taken a snapshot of ILC2 diversity. Therefore, diversity among ILC2s should be considered in future investigations. It is expected that future research exploring the heterogeneity of ILC2s will shed light on a variety of life science mysteries.

## Author Contributions

MA and HK screened articles related to this topic and wrote the manuscript. KF reviewed the manuscript critically for important intellectual content. All authors contributed to the article and approved the submitted version.

## Funding

The work is supported by Keio University Academic Development Funds, Keio Gijuku Fukuzawa Memorial Fund for the Advancement of Education and Research, The Mochida Memorial Foundation for Medical and Pharmaceutical Research, KAKENHI, Grant-in-Aid for Young Scientists grants JP20K17193 (HK).

## Conflict of Interest

The authors declare that the research was conducted in the absence of any commercial or financial relationships that could be construed as a potential conflict of interest.

## Publisher’s Note

All claims expressed in this article are solely those of the authors and do not necessarily represent those of their affiliated organizations, or those of the publisher, the editors and the reviewers. Any product that may be evaluated in this article, or claim that may be made by its manufacturer, is not guaranteed or endorsed by the publisher.
